# Correlation between Surgeon's Experience, Surgery Complexity and the Alteration of Stress Related Physiological Parameters

**DOI:** 10.1371/journal.pone.0112444

**Published:** 2014-11-07

**Authors:** Massimo Marrelli, Stefano Gentile, Francesca Palmieri, Francesco Paduano, Marco Tatullo

**Affiliations:** 1 Unit of Maxillofacial Surgery, Calabrodental, Crotone, Italy; 2 Tecnologica Research Institute, Biomedical Section, Crotone, Italy; University of North Carolina at Chapel Hill, United States of America

## Abstract

**Introduction:**

In the present work we analyzed the hormonal (salivary Cortisol; sC), immune (salivary Immunoglobulin A; sIgA) and cardiovascular (Heart rate, HR, and systolic blood pressure, SBP) responses induced by stress conditions in oral surgeons, randomly recruited according to their expertise level.

**Materials and methods:**

Each surgeon performed three different surgical procedures with increasing degrees of technical difficulty and under time-limited conditions, to assess whether these variants may influence the risks of stress-induced secondary hypertension among the involved health professionals. sC and sIgA samples and cardiovascular function measurements were taken up before, during, and two hours after every surgery. Salivary samples and cardiovascular measurements were taken also during non-surgical days, as baseline controls.

**Results:**

We observed that more experienced surgeons showed a higher stress management ability compared to those with less experience or, generally, younger, which are more exposed to the risks of developing secondary hypertension. Nevertheless, indipendently of sex and experience, oral surgeons are constantly exposed to high risks of developing stress-related diseases.

**Conclusions:**

On the basis of the issues addressed and the results obtained, we have highlighted the importance of the investigated stress biomarkers to monitor and to prevent stress-related pathologies among oral surgeons. This approach is aimed to emphasize the significance of these specific stress-biomarkers, which represent a powerful instrument to evaluate stress levels in oral surgeons, and that may help to reduce the most severe life-threatening risks to which they are daily exposed. In conclusion, final goal of this study is to suggest an useful guideline to monitor the stress levels of oral and maxillofacial surgeons in order to improve their quality of life, which is inevitably reflected on the quality of the performances provided and, finally, to prevent possible mistakes in their daily activities.

## Introduction

**Figure 1:**
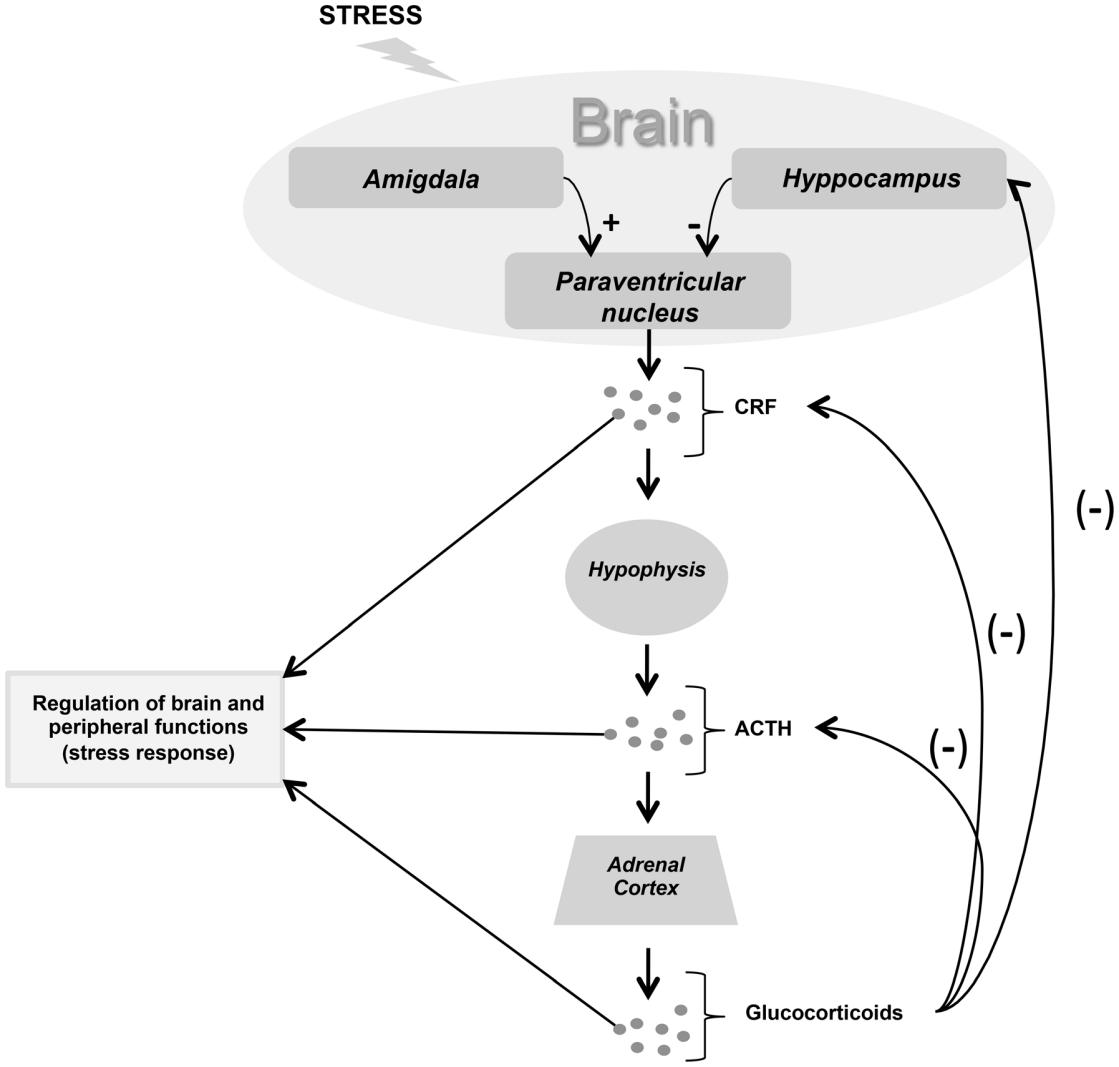
Schematic representation of the hypothalamus-hypophysis-adrenal cortex axis. Limbic system modulates the neuroendocrine response to emotional stress, such as occupational stress. The hypothalamus releases CRF which induces the synthesis of ATCH in the anterior lobe of the hypophysis. ACTH acts on adrenal cortex which produces glucocorticoids (manly cortisol in humans). Circulating Cortisol gives its negative feedback on hypothalamus and hypophysis. CRF (Corticotropin releasing factor), ACTH (Adrenocorticotropic-hormone).

Nowadays the concepts of stress are more complex and distinguish at least two broad categories of stressors: physical (systemic or reactive) and psychological (emotional or processing), with marked differential brain processing. Much attention has been paid to two physiological systems the hypothalamus-pituitary-adrenocortical axis, with the secretion of the glucocorticoid cortisol (**Fig. 1**), and the sympathetic-adrenomedullary system which induces the secretion of catecholamines and cardiovascular changes. These two systems are important not only because of their involvement in pathologic consequences of stress (e.g. depression, cardiovascular diseases, and suppression of the immune system), but also because they seem to reflect the intensity of stressful situations as seen from the results of animal investigations [1]. More precisely, the exposure to emotionally stressful situations that differ in intensity have been found to cause gradual increases in plasma levels of glucocorticoids, adrenaline and noradrenaline, and prolactin [2]–[4]. Salivary levels of cortisol, alpha-amylase, IgA and chromogranin A (CgA) [5]–[7], are also associated with stress state; for example, such parameters result significantly altered during several dental therapies [8].

Some stressors, such as prolonged mental and physical effort, public speaking, examinations, apprehension before surgery [8], [9], can induce an increasing of these stress biomarkers, with pathological consequences on health. One of the most common stressors which workers are often subjected to is the so called “occupational stress”. It is defined as the sum of physical, mental, and physiological responses to work which, when intensified, are transformed into negative emotional reactions that reflect into loss of productivity and reduction in the quality of the service offered [10]. The aspects of the work environment that often induce stress are: “practice demands and organization” and “lack of variation and perspective in work” [11]. These factors can lead to the development of the burnout syndrome, defined as the triad of high emotional exhaustion, high depersonalization, and low personal accomplishment [12].

A particular class of workers, the health professionals, is subjected to higher levels of occupational-stress than the average workers. It is well known that surgical procedures are mentally and physically demanding, and that a stress state during surgery may cause surgical mistakes that can compromise patient safety. Due to the high responsibility level of their work, surgeons, especially maxillofacial and oral surgeons [11]–[14], are more exposed to psychological and physiological stress outcomes such as depression and mental disorders, secondary hypertension and cardiovascular diseases, impairment of immune system, obesity, metabolic disorders and oxidative stress, and higher risk of developing neurodegenerative diseases such as Alzheimer's disease [15]–[19].

Mental and environmental factors may play a critical role in the stress state of surgeons; bad peri-operative sleep quality [20], rather than personal accomplishment, engagement levels and emotional exhaustion [11], [13], [14], have been demonstrated to be common stressors leading to burnout syndrome exacerbation. Beside the psychosocial aspects, there are several biological markers that should be kept under control to prevent stress physiopathological outcomes.

Some authors observed the alterations of specific stress biomarkers such as sC secretion, and HR variations [1], [21], in surgeons performing surgeries. These studies demonstrated that it is crucial to monitor such biomarkers to prevent the diseases typically induced by prolonged and repetitive stress states.

In the light of these findings, the aim of the present work is to analyze the above described biomarkers in three groups of oral surgeons, recruited according to their experience level, and engaged in three different surgical procedures with increasing degrees of technical difficulty, to assess whether these variants may influence hormonal/immune response (sC and sIgA) and cardiovascular response (HR and SBP) and consequently, whether they may affect the risk of developing clinical conditions such as secondary hypertension or, however, an overall decay of the general health status.

The final goal of this study is to suggest an useful guideline to monitor the stress levels of oral and maxillofacial surgeons in order to improve their quality of life, which is inevitably reflected on the quality of the performances provided and, finally, to prevent possible mistakes in their daily activities. This work is aimed to provide a valuable contribution for the improvement of the national health care service.

## Materials and Methods

All surgeons involved in this study gave signed informed consent following the guidelines approved by Calabrodental dental clinic (Crotone, Italy). The study and the related procedures were conducted in compliance with guidelines approved by the Calabrodental Ethics Committee. Calabrodental ethics committee specifically approved this study. The study was conducted in compliance with the "Ethical principles for medical research involving human subjects" of Helsinki Declaration. The study was conducted in accordance with Italian laws and regulations.

Surgeons were randomly grouped, according to their experience level, in three categories: *senior*, with more than 10 years of experience (N = 5, M/F = 4/1, mean age: 52±2); *expert*, between 5 and 10 years of experience (N = 5, M/F = 3/2, mean age: 38±3); *junior*, with less than 5 years of experience (N = 5, M/F = 2/3, mean age 30±3). We also classified the type of surgical procedures in three categories of increasing technical difficulty: *easy* (e.g. simple extraction surgery); *intermediate* (e.g. routine implantology); *complex*, which includes procedures of objective difficulty (e.g. sinus surgery and multiple implants insertion), and procedures of subjective difficulty (e.g. oral cysts removal or apicectomy). Each procedure has been performed in a range of limited narcosis time (less than two hours), and under complete free management of narcosis time duration.

### sC and sIgA assay

Salivary samples were collected in three different moments of the day. According to cortisol circadian cycle, samples were taken in the morning (7:30–8:00 am, T_1_), during surgery (10am–1pm, T_2_), and two hours after surgery (T_3_). Saliva has been collected using a *Salivette swab* which allows collect 1 mL of saliva. For each operator two control samples have been collected in a non-surgical day, at two different time points, to draw a baseline (BL). The same protocol has been used for salivary IgA. Each surgeon has been checked for the eventual presence of upper respiratory tract infection (URTI). We used sC and sIgA ELISA kit (Salimetrics, Pennsylvania, USA), while saliva samples were treated according to the protocols previously described by Papadopoulos et al. [22].

### HR and SBP measurements

Each surgeon involved in the study was measured HR and SBP in three different moments: 1) at the arrival in the workplace (7:30–8:00am, T_1_); 2) during the surgical procedure (10am–1pm, T_2_); 3) two hours after surgery (T_3_). Each measurement has been taken twice so that every value has been expressed as mean ± SD (standard deviation). Similarly to sC and IgA, we took two HR and SBP measurement in a non-surgical day (BL) at two different time points.

### Physiological values

sC concentrations in the range of 2.3–12.7 ng/mL (6.35–35.05 nmol/mL) in the morning, and 0.5–4.2 ng/mL (1.38–11.60 nmol/mL) after lunch were considered physiological. sIgA physiological range was assessed around 20–50 ng/mL, while SBP and HR were assessed at 120 mmHg and 60–70 bpm respectively. Results were expressed as mean ± standard deviation (SD).

## Results

### sC and sIgA levels in senior, expert and junior surgeons performing an easy surgical procedure

In T_1_ sC levels were significantly higher in *expert* surgeons (5.6±0.6 ng/mL), compared to those of *senior* and *junior* surgeons (3.2±0.4 and 3.8±0.4 ng/mL respectively) remaining, however, within a physiological range. *Senior* surgeons showed a slightly increase of sC in T_2_, which decreased back to BL value in T_3_ (**Tab. 1**) outlining a bell shaped trend. Apart from the significant increase in T_1_, *expert* surgeons showed physiological sC values in T_2_ and T_3_ although still higher compared to BL (**Tab. 1**). Interestingly, *junior* surgeons had a significant increase of sC in T_3_ (5.8±0.8 ng/mL) which reflected the cardiovascular response observed at the same check point. However, in all three groups we didn't detect critical levels of sC, which is associable with the relative easyness of the surgical procedure. Similarly, sIgA assay results from all three surgeons groups didn't show any significant alterations, remaining within physiological range in T_1_, T_2_ and T_3_ (**Tab. 2**). Unexpectedly, limited time conditions didn't affect hormonal nor cardiovascular stress-responses in all three groups, regardless of the difficulty of the surgery (as well as for intermediate and complex procedures; data not shown).

**Table 1:** sC baselines compared to sC concentrations registered in T_1_, T_2_ and T_3_ of each specific surgical procedure.

Salivary Cortisol (ng/mL)												
Surgical procedure difficulty												
	*Easy*				*Intermediate*				*Complex*			
	BL	T_1_	T_2_	T_3_	BL	T_1_	T_2_	T_3_	BL	T_1_	T_2_	T_3_
**Senior**	3.5±0.7	3.2±0.4	4.3±0.4	3.6±0.2	3.5±0.7	3.4±0.1	6.5±0.3	4.4±0.1	3.5±0.7	3.8±0.1	5.9±0.3	4.1±0.1
**Expert**	3.2±1.6	5.8±0.6	4.7±1.3	4.3±0.8	3.2±1.6	9.6±1.2	7.1±0.8	5±1.4	3.2±1.6	13.5±2.3	6.4±0.6	3±0.7
**Junior**	3.6±0.9	3.8±0.4	3.3±0.5	5.8±0.8	3.6±0.9	5.5±0.8	4.7±0.8	5.6±0.6	3.6±0.9	7.1±1.1	6.5±0.8	4.3±0.4

Each value is expressed as the mean ± SD. BL = baseline; T1 = before surgery (7:30/8:00 am); T2 = during surgery (10:00am/1:00pm); T3 = post surgery (2 h after surgical procedure).

sC: Salivary cortisol.

**Table 2:** sIgA baselines compared to sIgA concentrations registered in T_1_, T_2_ and T_3_ of each specific surgical procedure.

Salivary IgA (ng/mL)												
Surgical procedure difficulty												
	*Easy*				*Intermediate*				*Complex*			
	BL	T_1_	T_2_	T_3_	BL	T_1_	T_2_	T_3_	BL	T_1_	T_2_	T_3_
**Senior**	49±2.2	46±0.8	±52±1.3	48±3.7	49±2.2	48±4.8	50±1.3	45±4.1	49±2.2	50±3.0	51±2.7	50±0.2
**Expert**	38±1.6	47±2.3	44±2.3	36±4.1	38±1.6	46±2.1	39±1.1	41±0.2	38±1.6	48±4.1	40±3.9	43±1.4
**Junior**	47±3.4	45±3.3	43±0.7	45±3.2	47±3.4	45±1.3	45±0.8	46±1.4	47±3.4	47±0.4	46±1.6	46±2.1

Each values is expressed as the mean ± SD. BL = baseline; T1 = before surgery (7:30/8:00 am); T2 = during surgery (10:00am/1:00pm); T3 = post surgery (2 h after surgical procedure). sIgA: Salivary Immunoglobulin A.

### HR and SBP in senior, expert and junior surgeons performing an easy surgical procedure

Cardiovascular parameters analysis showed no significant or pathological alterations of HR and SBP in all three groups. More precisely, all surgeons examined kept their cardiovascular values within physiological range with a normal HR in T_1_, T_2_ and T_3_, and slight increases of SBP only in T_2_ for *senior* and *expert* groups (**Tab. 3**), and in T_3_ (130±2.2) only in *junior* surgeons group.

**Table 3:** HR and SBP baselines compared HR and SBP registered in T_1_, T_2_ and T_3_ of each specific surgical procedure.

HR (beats/min) and SBP (mmHg)												
Surgical procedure difficulty												
	*Easy*				*Intermediate*				*Complex*			
	BL	T_1_	T_2_	T_3_	BL	T_1_	T_2_	T_3_	BL	T_1_	T_2_	T_3_
**Senior HR**	65±2.4	70±3	75±2.6	71±2.8	65±2.4	72±1.3	83±2.8	71±2.4	65±2.4	71±2.4	84±2	70±2.4
**Senior SBP**	122±1.3	125±0.8	128±2.7	122±1.5	122±1.3	128±2.4	144±1.7	128±1.7	122±1.3	122±1.3	131±2.5	123±3
**Expert HR**	69±4	77±1.8	79±2.5	77±3.8	69±4	88±2.3	82±3.4	80±2.5	69±4	104±4	95±1.5	70±2
**Expert SBP**	118±2.3	123±2	126±1.1	120±1.5	119±2.3	142±1.6	135±2.3	116±3	119±2.3	140±1.9	136±1.9	127±1.7
**Junior HR**	70±5.4	73±4	76±2.4	80±4.3	70±5.4	78±2.9	83±2.3	83±6.4	69±4	89±2.5	102±6.7	100±5
**Junior SBP**	119±1.6	124±1	126±1.9	130±2.2	119±1.6	124±2.4	133±3.2	129±2.2	119±1.9	138±2.3	146±2.6	131±1.6

Each value is expressed as the mean ± SD. BL = baseline; T_1_ = before surgery (7:30/8:00 am); T_2_ = during surgery (10:00am/1:00pm); T_3_ = post surgery (2 h after surgical procedure). HR: Heart rate. SBP: Systolic Blood Pressure.

### sC and sIgA levels in senior, expert and junior surgeons performing an intermediate surgical procedure

In this case we started to record some values that could be considered para-physiological. *Senior* surgeons' sC fluctuations maintained the regular bell shaped trend observed so far, increasing only in T_2_ (6.5±0.3 ng/mL). *Expert* surgeons showed significantly high sC levels in T_1_ and T_2_ (9.6±1.2 and 7.1±0.8 ng/mL respectively), corroborating the hypothesis of their hyper susceptibility during pre-surgery, while *junior* surgeons' sC levels have remained constantly higher compared to their BLs, but still within physiological range, towards T_1_, T_2_ and T_3_ (**Tab. 1**). On the other hand, sIgA variations weren't significant in any of the groups examined (**Tab. 2**).

### HR and SBP in senior, expert and junior surgeons performing an intermediate surgical procedure

With the increase of the difficulty level of the surgical procedures, we observed some significant alterations of the physiological cardiovascular homeostasis. Once again, *senior* surgeons' cardiovascular response reflected the trends we've observed until now, with the classic bell shaped curve characterized by slightly alterations of HR and SBP in T_1_ and T_3_ (**Tab. 3**), and a marked SBP increase only in T_2_ (144±4 mmHg). *Expert* surgeons instead, confirmed their hyper-anxiety in T_1_, manifesting considerable SBP and HR peaks (142±1.6 mmHg and 88±2.3 beats/min). Interestingly, *junior* surgeons showed a surprisingly good stress management in a situation of intermediate difficulty level, with HR and SBP values within physiological range towards T_1_, T_2_ and T_3_ (**Tab. 3**).

### sC and sIgA levels in senior, expert and junior surgeons performing a complex surgical procedure

On the base of the data obtained, we observed that *senior* surgeons showed an appreciable regularity of sC fluctuations even during complex surgical procedures (**Tab. 1**), displaying a remarkable ability to manage stress. On the other hand, sC values registered in T_1_ and T_2_ for *expert* and *junior* surgeons (**Tab. 1**) suggest that these two categories suffered particularly pre- and surgical stress. We may speculate that these types of surgical procedures (e.g. sinus surgery, multiple implants, cystectomy and apicectomy), that require a great deal of mental and physical effort, induce a higher stressful response in surgeons with less than 10 years of experience compared with more experienced ones. In this analysis, stress in T_1_ was significantly higher in *expert* (13.5±2.3 ng/mL) than in *junior* (7.1±1.1 ng/mL) surgeons, whose sC values were anyhow beyond the physiological range. This could be due to subjective factors, accounting the possibility that *expert* surgeons may somatize more than *junior* surgeons, or it could be a matter of higher awareness of *expert* surgeons about the possible risks of the surgical procedure. In T_2_, sC levels of *senior*, *expert* and *junior* surgeons were similar (**Tab. 1**), however, considering the sC levels showed in the T_1_ (**Tab. 1**), we hypothesized that *senior* surgeons had a more controlled approach to T_1_, and that the increase of sC in T_2_ may have been due to higher mental effort demand rather than stress *per se*. Notably, *Expert* surgeons showed a significant decrease of sC in T_3_, probably caused by a physiological-adaptive response. Even in this case, sIgA levels weren't significantly altered in any group examined (**Tab. 2**).

### HR and SBP in senior, expert and junior surgeons performing a complex surgical procedure

*Senior* surgeons displayed an even more effective stress management than that observed during intermediate surgical procedure. This could be explained by the fact that these surgeons, due to their long career and to their high skills and preparation levels, are more confident with more difficult and challenging surgical procedures that they perform more frequently than *expert* and *junior* surgeons. In terms of alterations of cardiovascular parameters, *senior* surgeons showed the typical bell shaped curve, with HR and SBP fluctuations within the upper physiological range (**Tab. 3**). *Expert* surgeons, on the other hand, showed a mild tachycardia in T_1_ and T_2_ together with significant SBP increase (**Tab. 3**), whereas HR and SBP in *junior* surgeons remained high towards T_1_, T_2_ and T_3_ (**Tab. 3**) showing mild tachycardia and reaching SBP values close to the upper cut-off limit of the physiological range (146±2.6 mmHg). These data basically reflect how, aside from the specific physical and emotional characteristics of each subject examined, experience level of the surgeon affects the ability to control the cardiovascular abnormalities in response to a high stress level resulting from a high mental and physical effort demanding situation such as a complex surgical procedure.

## Discussion

It's generally reported in the literature that dentists, particularly oral surgeons, have been singled out as the health care professionals more likely to be subjected to severe stress burnout [12]. Many authors have analyzed the intricate psychosocial implications, derived from the stress conditions to which surgeons are daily subjected to [11]–[14], [20], highlighting the main work-environmental aspects of stress. In the present work we examined the variations of biological parameters of hormonal (sC), immune (sIgA) and cardiovascular (HR and SBP) responses to occupational stress. Over the last 30 years, there has been an increasing appreciation of the clinical utility for the measurement of sC [23]. Although it has been demonstrated that people undergoing stress/anxiety state [6], particularly during dental surgery [5], showed high sC concentrations, to our knowledge only few works have measured sC levels among surgeons engaged in surgical procedures [21], and yet none among oral surgeons performing oral surgery procedures. In our study, surgeons were classified in three groups according to their experience level (*senior*: more than 10 years of experience; *expert*: between 5 and 10 years of experience; *junior*: less than 5 years of experience). Each group performed three different types of surgical procedures (complex, intermediate, easy) under two time conditions: limited narcosis time (less than 2 hour), and complete free management of the duration of narcosis.

We've collected sC, according to its circadian cycle [23], in three different moments of the day as indicated in the materials and methods session (T_1_, T_2_ and T_3_). We observed that the general trend among the three classes of surgeons was an increase of sC in T_1_ and T_2_, and a subsequent decrease in T_3_, with peculiar characteristics per group. More precisely *senior* surgeons showed a remarkably stress management ability independently of the difficulty levels of the surgical procedure, whereas *expert* surgeons, rather than *junior*, showed quite high concentrations of sC in T_1_, independently of the difficulty of the procedure. We hypothesized that *expert* surgeons, being half way between *senior* and *junior*, have not yet gained the confidence of the first, but are more aware of the possible risks of surgical failure compared to *junior*, thus this would induce the soaring increase of sC. Nevertheless, sC concentrations remained higher within surgical days compared to BL among all three groups.

The link between mental stress, cortisol reactivity, and hypertension development risk it has been shown to be plausible [24] because cortisol can directly influence the central nervous system, affecting those brain's areas involved in the control of blood pressure (i.e. hypothalamus, limbic system). In addition, glucocorticoids receptors are present in the heart and in the smooth muscle cells of the resistance vessels [25].

Similarly to cortisol, salivary immunoglobulin levels are known to increase in association with coronary and artery disease [26], and local and systemic immunologic responses to stress conditions [27]. Particularly, sIgA has been shown to be increased under mental stress conditions [28].

Contrary to what it was stated above, we found no significant alterations of sIgA levels in all three surgeon groups. Our findings are consistent with what other authors observed in elite basketball players playing a basketball match [29], and in young swimmers during competition week [22].

Comparing these two results (sC and sIgA levels variations), we evicted that sC levels fluctuations are more sensitive than sIgA level variations to the different stress conditions to which surgeons, involved in the present study, were subjected. Moreover, sC variations better correlate with the cardiovascular variations we observed. It is therefore reasonable to point out sC as a better stress-related biomarker, compared to sIgA, to keep under control to prevent stress-induced cardiovascular disease development.

Together with the analysis of the above salivary biomarkers, we monitored the main cardiac parameters (HR and SBP). Previous studies [1], [21], [30] have investigated the cardiovascular changes in surgeons during surgical procedures. These works showed that HR was higher in novice surgeons, rather than more experienced surgeons, before and during the surgical procedure, decreasing in the post-surgery. We observed a similar trend for HR and SBP, with specific peculiarity within each group examined. More precisely, cardiovascular alterations followed a regular bell shaped curve in the *senior* group towards T_1_, T_2_ and T_3_, whereas *expert* surgeons response was characterized by a marked increase of HR and SBP in T_1_ which decreased to physiological values in T_2_ and T_3_, lastly, *junior* surgeons generally kept their HR and SBP values high towards T_2_ and T_3_ showing lesser stress management ability compared to *senior*. Furthermore, we noticed that the difficulty of the surgical procedure influenced the cardiovascular alterations, as well as sC secretion, in *expert* and *junior* surgeons more significantly than in *senior* surgeons, while narcosis duration conditions didn't affect significantly both hormonal and cardiovascular responses in all three groups.

The present work showed that *senior* surgeons display a better stress management capacity, letting stress to increase only when mental and physical demand reach its peak during the performance of surgical procedures, and keeping sC, sIG and HR and SBP fluctuations constant despite the difficulty level of the surgical procedure, thereby giving a pure data which is not entirely influenced by the specific context. Another interesting aspect is that *senior* surgeons are able to keep stress at minimum levels during non-surgical days, maintaining the BL not too far from physiological range. Nevertheless, we constantly observed alterations of the physiological cardiovascular function during surgical days, compared to the BLs, among all three groups, thus indicating, in almost all cases we examined, a persistent stress condition which keeps blood pressure straddling the edge between physiological and hypertensive. This indeed reflects the daily increase of physical and mental effort demand, corroborating the concept that oral surgeons are constantly exposed to high risk of developing secondary hypertension, especially in the case of long year careers.

## Conclusions

**Figure 2:**
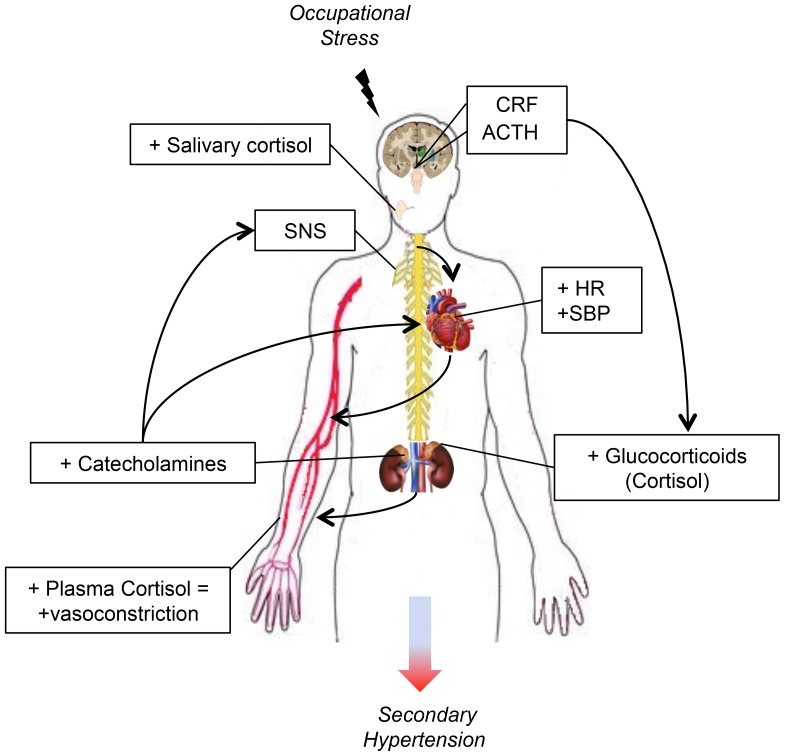
Biological mechanisms involved in the development of occupational stress-induced secondary hypertension. CRF (Corticotropin releasing factor), ACTH (Adrenocorticotropic-hormone), SNS (Sympathetic nervous system), HR (Heart rate), SBP (Systolic blood pressure).

In conclusion we observed that oral surgeons, independently of the surgical experience and gender, are exposed to higher risks of developing stress-related secondary hypertension (**Fig. 2**), thus it is of relevant importance to implement new strategies of prevention and monitoring of the above investigated stress biomarkers, as well as more effective methods that help oral surgeons to keep stress down before and during a surgical procedures such as mental practice (MP) [21], [31]. Our work aims to involve Government, National and International Agencies' attention, in order to reduce life-threatening risks, to which these health professionals are daily exposed, thus to safeguard surgeons' wellbeing and to identify and minimize those factors that may cause the occurrence of errors during surgical procedures and, more generally, negatively affect the quality of the health care service.
